# Association between leisure time physical activity preference and behavior: evidence from the China Health & Nutrition Survey, 2004–2011

**DOI:** 10.1186/s12889-017-4386-5

**Published:** 2017-05-16

**Authors:** Junmin Zhou, Denise H Britigan, Shireen S. Rajaram, Hongmei Wang, Dejun Su

**Affiliations:** 1 West China School of Public Health, Sichuan University, Chengdu, 610041 China; 2 Department of Health Promotion, Social & Behavioral Health, College of Public Health, University of Nebraska Medical Center, Omaha, NE 68198 USA; 3 Department of Health Services Research & Administration, College of Public Health, University of Nebraska Medical Center, Omaha, NE 68198 USA

**Keywords:** China, China Health & Nutrition Survey, Leisure time physical activity, Preference and behavior, Urban and rural disparities

## Abstract

**Background:**

Previous studies have suggested that food preference is a good indicator of actual food intake and that sedentary activity preference is a significant predictor of lower physical activity level. But no studies have examined the direct relationship between leisure time physical activity (LTPA) preferences and actual LTPA behavior, especially studies using longitudinal data. This study seeks to determine the association between these two variables, and to assess whether the association differs between urban and rural areas in China.

**Methods:**

A total of 2427 Chinese adults were included in the analysis. Spearman correlation coefficients were used to test the association between leisure time physical activity preference and behavior, followed by multiple logistic regressions to further examine the association after adjusting for possible confounding variables. Urban-rural differences in the association were investigated through stratified analysis.

**Results:**

In the sample, 63.0% were from urban areas, 47.4% were men, and the mean age was 40. Adjusted estimates based on logistic regression show that LTPA preference was a significant predictor of actual LTPA behavior (OR = 1.05, 95% CI = 1.01–1.09). The correlation was found to be significant among urban residents (OR = 1.06, 95% CI = 1.01–1.10), but not in rural residents.

**Conclusions:**

The study illustrates the predictive value of LTPA preference for actual LTPA behavior. Changing LTPA preference to promote LTPA may be helpful in preventing and controlling chronic disease in China.

## Background

Findings from a number of studies have suggested that food preference is a good indicator of actual food intake and is also found to be associated with cardiovascular disease [1–4]. Exploring the association between physical activity preference and physical activity behavior thus appears to be promising and necessary. Studies on sedentary activity preference indicate that it is a significant predictor of lower physical activity level [5, 6], and preferences for physical activity and psychological variables such as anxiety, depression, and avoidance explained significant physical activity changes among children [7]. To the best of our knowledge, however, no studies have examined the direct relationship between preference for LTPA and actual LTPA behavior in adults. To further understand the determinants and predictors of physical activity behavior, it becomes necessary to investigate the association between LTPA preference and behavior, especially by using longitudinal data to examine causal effects.

Such a step is important in consideration of the complexity and difficulty in reliable measurements of LTPA. Comprehensive and relevant measurement of LTPA is fundamental to health promotion [8]. It can be challenging, however, to obtain accurate and reliable measurements of LTPA. One major issue relates to whether LTPA is subjectively or objectively assessed. There is evidence that correlations between self-reporting measures of LTPA and direct measures were low to moderate, suggesting that measurement methods may have a substantial impact on the observed level of LTPA [9]. This finding points to the importance of identifying alternative measures of LTPA, including LTPA preference.

In China, LTPA level is low and with recent, continuous evidence of decrease. Data from the China Health & Nutrition Survey shows that LTPA in Chinese adults has dropped from 382MET-h/week in 1991 to 264MET-h/week in 2011 [10]. Examining the association of LTPA preferences and association to actual behavior would provide valuable information for the design of future interventions that focus on preference education as one means to reverse this alarming trend. In addition, a growing number of studies have been focused on the disparities between urban and rural residents in LTPA in China. Specifically, urban adults were more physically active than their rural counterparts during leisure time [11]. Urban adults exercised more regularly, whereas a considerable increase was seen in rural residents who owned televisions [12, 13]. However, there is no evidence showing that LTPA has increased significantly in either urban or rural residents during the last several decades. In consideration of the substantial urban-rural disparities in China in terms of occupational structure, culture, access to recreational and exercise facilities, and the like, this study seeks to assess the association between LTPA preference and behavior in urban and rural residents by conducting separate stratified analyses.

We hypothesized that LTPA preference is predictive of actual physical activity behavior, after adjusting for likely confounding variables. A secondary hypothesis is that, due to the strong degree of labor-intensive farming by rural Chinese, the association between LTPA preference and actual LTPA behavior would be less apparent in rural China than it would in urban China.

## Methods

### Data

The source of data comes from the China Health and Nutrition Survey (CHNS). CHNS, an ongoing open cohort and international collaborative project between the Carolina Population Center at the University of North Carolina at Chapel Hill and the National Institute of Nutrition and Food Safety at the Chinese Center for Disease Control and Prevention, was designed to examine the effects of the health, nutrition, and family planning policies and programs implemented by national and local governments. The focus of CHNS is how the social and economic transformation of Chinese society is affecting the health status of its population. The study took place over three days, using a multistage, random cluster process to draw a sample of about 4400 households with a total of 26,000 persons who live in nine provinces that vary substantially in geography, economic development, public resources, and health indicators.

**Fig. 1:**
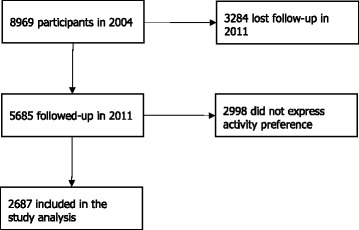
Sample included in the analysis

The data used in this study comes from Year 2004 and Year 2011 in CHNS. As shown in Fig. 1, there were 8969 adults in 2004 and 12,235 in 2011, with 5685 participants participating both years. Of those 5685 participants, 2998 did not report preferences on physical activities in 2004, so the final sample size for data analysis was 2687. The attrition of samples is large because of substantial migration out of rural China since the 1980s, and the young, aged from 15 to 40, make up a large proportion of participants that attrited from the study between 2004 and 2011 [14]. Nevertheless, studies suggested that the potential bias associated with attrition should not be a serious concern, because among large-scale surveys in developing countries, the CHNS is one of the most successful longitudinal studies in keeping attrition low [15, 16]. Table 3 in the Appendix compares participants with and without physical activity preferences, with no substantial differences observed between them.

The data used in the study is the secondary data from CHNS. The data contains no identifiers and is accessible to and may be downloaded by the public. All authors have received necessary ethics training and relevant certificates (such as Collaborative Institutional Training Initiative) to work on human sciences.

### Measurements

#### LTPA preference

In the CHNS questionnaire, respondents were asked regarding each activity if they: like very much, like somewhat, are neutral, dislike somewhat, or dislike very much, with the physical activities listed being: Walking and Tai Chi, Sports (ping pong, badminton, tennis, soccer, basketball, volleyball), and Body building. The grading scale for LTPA preference was adapted from a previous study [17].

Specifically, the extent to which participants liked or disliked a certain type of physical activity was rated on a scale of 1–5, with 1 indicating strongly dislike of the physical activity and 5 indicating liking the physical activity very much. Next, participants’ physical activity preference was determined by adding all three scores (Walking and Tai Chi; Sports; Body building). Thus, the range of physical activity preference scores for each participant falls between 3 and 15. The reliability of the Physical Activity scale using Cronbach’s alpha coefficient was 0.70, suggesting good internal consistency.

#### LTPA behavior

In the CHNS, participants were asked to answer the questions, “Do you participate in this activity?” with the choices: “yes,” “no,” and, “unknown.” If “yes” was selected to a given item, respondents were then asked to further indicate how much time they spent on the activity during a typical weekday or weekend day. For each type of activity, the average time per day was calculated (“0” given for those who did not participate): Average time = (Time spent on each workday*5 + Time spent on each weekend day*2)/7. Then, the total average time of physical activity per day was determined by averaging scores of the 3 activities. This measurement for physical activity behavior is similar to that of the Older Adult Exercise Status Inventory study [18, 19] that has strong validity and test-retest reliability [20, 21]. Validity and test-retest reliability of the LTPA measure used in this study should thus be met. LTPA measures were calculated in both year 2004 and 2011.

#### Potential confounding variables

Potential confounding variables included sociodemographic variables, health behaviors, and health-related variables. Sociodemographic variables include age, sex, ethnicity, marital status, community types (urban vs. suburban vs. town vs. village), region of residence (north vs. south), education, employment status, and annual household income (Chinese Yuan Renminbi - RMB: ¥; Yuan-US Dollar exchange rate was 6.4588 Yuan per U.S dollar in 2010; annual household income was grouped into four levels according to quartiles: 0–8000, 8001–15,000, 15,001–25,000, and over 25,000). Region of residence was divided into north and south based on Huai River policy, because 5.5 years of disparity in life expectancy between north and south China had been observed in a previous study [22]. Health behavior variables included smoking status and alcohol consumption. Health-related variables included current health status (self-reported), health insurance coverage, and Body Mass Index (BMI). A unique BMI criterion was applied, recognizing that Chinese have different body shapes and skeletons compared to Westerners: a growing number of studies reveals that Chinese and several other population in Asian Pacific countries have increased risk for obesity-related chronic diseases or conditions at a lower BMI than do Westerners [23–28]. Using this adapted scale, underweight is <18.50, normal weight is 18.50–23.99, overweight is 24.00–27.99, and obesity is 28.00 and over [29].

### Statistical analysis

A univariate analysis was conducted to depict the distribution of all explanatory and control variables. Spearman Correlation Coefficients were used to test associations between physical activity preference in 2004 and physical activity behavior in 2004 and 2011.

The association between physical activity behavior in 2011 and physical activity preference in 2004 was also assessed by conducting multivariate logistic regressions, adjusting for age, sex, ethnicity, marital status, community types, region of residence, education, employment status, annual household income, smoking status, alcohol consumption, current health status (self-report), health insurance coverage, and BMI category. All covariates were from data collected in 2004. To explore potential urban and rural differences, analyses were also conducted separately for urban residents and rural residents. To test for possible confounding effects through BMI and current health status (self-report), models were run with and without these two variables. Odds ratios (ORs) and 95% confidence intervals (95% CIs) were reported. The association was considered to be statistically significant if the 2-sided *p* value is less than 0.05. All analyses were performed using SPSS for Windows, version 21.0 [30].

## Results

Table 1 shows descriptive statistics for all outcome variables and covariates in the whole sample, for urban residents, and for rural residents. Overall, the prevalence of leisure-time physical activity in 2011 was low in our sample (14.1%), and it was much more prevalent among urban residents (20.2%) than their rural counterparts (4.0%). The LTPA preferences of the participants were low in 2004 (mean preference score was 6.16 in the whole sample, and again, the possible preference score is between 3 and 15). Urban residents were more likely than rural citizens to report higher LTPA preference (6.58 vs. 5.45). In ethnic composition of the study, the Han dominated our sample (almost 90% of respondents were of Han ethnicity across different community groups), which is consistent with the national ethnic distribution in China. Urban residents were more likely to be widowed than rural participants (2.7% vs. 1.6%). Education level was low in our sample: specifically, less than one third (30.3%) of the sample received a high school education or higher. The disparity between urban and rural residents in education was substantial. Thirty nine point 2 % (39.2%) of urban residents reported high school or above education, compared to only 15.6% among rural residents. Over one-third (37.7%) of the whole sample was from a rural area (village), while 62.3% were from urban areas (including urban, suburban, and town). Urban residents were less likely to be smokers (31.7% vs. 34.1%), and more likely to drink alcohol (36.0% vs. 32.7%) than their rural counterparts. Urban residents also tended to have higher Body Mass Index (BMI): they specifically were less likely to be underweight (3.1% vs. 5.1%) or have normal weight (49.0% vs. 58.2%), and more likely to be overweight (37.7% vs. 29.3%) and obese (10.9% vs. 7.4%). Urban residents were also more likely to be covered by health insurance than rural people (43.6% vs. 22.3%).

**Table 1:** Variables Used in Analysis of Physical Activity Preference and Behavior in the Sample (*N* = 2427), Urban (*n* = 1528) and Rural (*n* = 899) Residents

	Whole Sample		Urban Residents		Rural Residents	
Variables	Number	Mean or Percentage (SD)	Number	Mean or Percentage (SD)	Number	Mean or Percentage (SD)
Dependent variable (2011)						
Physical activity						
No	2268	85.9	1313	79.8	955	96.0
Yes	373	14.1	333	20.2	40	4.0
Independent variables (2004)						
Physical activity preference						
Prefer activity	2638	6.16 (3.07)	1641	6.58 (3.17)	997	5.45 (2.77)
Demographics						
Age	2645	40.49 (12.84)	1648	42.35 (13.38)	997	37.42 (11.26)
Sex						
Male	1253	47.4	773	46.9	480	48.1
Female	1392	52.6	875	53.1	517	51.9
Ethnicity						
Han	2391	90.4	1523	92.4	868	87.1
Others	254	9.6	125	7.6	129	12.9
Marital status						
Never married	108	4.1	72	4.4	36	3.6
Married	2448	93.0	1513	92.3	935	94.3
Divorced	16	0.6	11	0.7	5	0.5
Widowed	60	2.3	44	2.7	16	1.6
Community types						
Urban	482	18.2	482	29.2	-	-
Suburban	640	24.2	640	38.8	-	-
Town	526	19.9	526	31.9	-	-
Village	997	37.7	-	-	997	100.0
Region of residence						
North	1268	47.9	747	45.3	521	52.3
South	1377	52.1	901	54.7	476	47.7
Socioeconomic status						
Employment						
Unemployed	570	21.6	479	29.1	91	9.1
Employed	2072	78.4	1167	70.9	905	90.9
Annual household income (Yuan)						
0–8000	520	19.9	242	14.8	278	28.4
8001–15,000	643	24.6	356	21.8	287	29.3
15,001–25,000	601	23.0	380	23.3	221	22.6
Over 25,000	850	32.5	656	40.1	194	19.8
Education						
Illiterate	464	17.6	233	14.2	231	23.2
Primary school	593	22.5	291	17.7	302	30.3
Middle school	781	29.6	473	28.8	308	30.9
High school or above	799	30.3	644	39.2	155	15.6
Health behavior						
Smoking status						
Nonsmoker	1781	67.4	1125	68.3	656	65.9
Smoker	861	32.6	522	31.7	339	34.1
Alcohol consumption						
No drinking	1726	65.3	1055	64.0	671	67.3
Drinking	919	34.7	593	36.0	326	32.7
Health-related variables						
BMI categories						
Underweight	96	3.8	49	3.1	47	5.1
Normal weight	1313	52.4	773	49.0	540	58.2
Overweight	855	34.1	583	37.7	272	29.3
Obese	241	9.6	172	10.9	69	7.4
Current health status (self-report)						
Very good	433	16.4	244	14.8	189	19.0
Good	1185	44.9	761	46.2	424	42.6
Bad	872	33.0	553	33.6	319	32.1
Very bad	151	5.7	88	5.3	63	6.3
Health insurance coverage						
No	1699	64.5	925	56.4	774	77.7
Yes	936	35.5	714	43.6	222	22.3

*Abbreviation: SD* standard deviation; —, not applicable

The Spearman correlation coefficients between LTPA behavior (in 2004 and 2011) and LTPA preference in 2004 shows that the time spent on LTPA in 2004 was positively and significantly related to their preference in the same year among the entire sample (coefficient = 0.202, *p* < 0.001), and among urban residents (coefficient = 0.201, *p* < 0.001) and rural residents (coefficient = 0.111, *p* < 0.001). However, the association was stronger in urban residents. Furthermore, the amount of time spent in physical activity in 2011 was positively and significantly associated with physical activity preference in 2004 among the whole sample (coefficient = 0.126, *p* < 0.001) and urban residents (coefficient = 0.111, *p* < 0.001), but not among rural residents.

Table 2 shows the multivariate logistic regressions assessing the association between LTPA behavior in 2011 and preference in 2004, after adjusting for age, sex, ethnicity, marital status, community types, region of residence, education, employment status, annual household income, smoking status, alcohol consumption, current health status (self-reported), health insurance coverage, and BMI. LTPA preference in 2004 was a significant predictor of LTPA behavior in the whole sample (OR = 1.05, 95% CI = 1.01–1.09) and urban sample (OR = 1.06, 95% CI = 1.01–1.10), but not among rural residents (OR = 1.04, 95% CI = 0.92–1.17). Age was also significantly associated with higher probability of physical activity in the whole sample (OR = 1.01, 95% CI = 1.00–1.03) and urban sample (OR = 1.02, 95% CI = 1.00–1.03). Married rural residents significantly related to lower probability of physical activity (OR = 0.12, 95% CI = 0.03–0.53) compared to their counterparts who never married. In the whole sample, village (OR = 0.30, 95% CI = 0.20–0.47) and town residents (OR = 0.69, 95% CI = 0.49–0.99) had a lower probability of physical activity. Participants in the whole sample and urban sample with higher education levels tended to be more involved in physical activity. Specifically, an elevated probability of physical activity, compared to participants who never attended school or could not read and write, was associated with those who had attended primary school (OR = 1.92, 95% CI = 1.14–3.25 in the whole sample; OR = 1.81, 95% CI = 1.02–3.22 in urban residents), middle school (OR = 2.53, 95% CI = 1.51–4.23 in the whole sample; OR = 2.46, 95% CI = 1.41–4.30 in urban residents), and high school or above (OR = 3.72, 95% CI = 2.22–6.22 in the whole sample; OR = 3.25, 95% CI = 1.86–5.68 in urban residents) as their highest education level. The associations were diminished in rural residents except for those who had high school or above education, who had a higher probability of physical activity (OR = 9.33, 95% CI = 2.20–39.55). Of note, participants covered by health insurance were more likely to report physical activity (OR = 1.77, 95% CI = 1.35–2.33 in the whole sample; OR = 1.53, 95% CI = 1.14–2.06 among urban residents; OR = 4.07, 95% CI = 1.92–8.60 for rural participants).

**Table 2:** Multivariate Logistic Regression on Physical Activity Behavior among Sample (*N* = 2427), Urban (*n* = 1528) and Rural (*n* = 899) Residents

Variables	Whole Sample (*N* = 2427)	Urban Residents (*n* = 1528)	Rural Residents (*n* = 899)
	Odds Ratio (95% CI)	Odds Ratio (95% CI)	Odds Ratio (95% CI)
Physical activity preference			
Prefer activity in 2004	1.05** (1.01–1.09)	1.06** (1.01–1.10)	1.04 (0.92–1.17)
Demographics			
Age	1.01** (1.00–1.03)	1.02** (1.00–1.03)	1.01 (0.97–1.05)
Sex			
Male	1 [Reference]	1 [Reference]	1 [Reference]
Female	1.23 (0.87–1.75)	1.25 (0.86–1.83)	1.37 (0.47–3.99)
Ethnicity			
Han	1 [Reference]	1 [Reference]	1 [Reference]
Others	1.26 (0.81–1.96)	1.18 (0.71–1.95)	1.87 (0.66–5.30)
Marital status			
Never married	1 [Reference]	1 [Reference]	1 [Reference]
Married	0.67 (0.37–1.24)	0.86 (0.43–1.69)	0.12*** (0.03–0.53)
Divorced	0.40 (0.08–2.13)	0.62 (0.11–3.50)	0.00 (0.00–0.00)
Widowed	0.64 (0.22–1.85)	0.73 (0.23–2.28)	0.22 (0.01–7.53)
Community types			
Urban	1 [Reference]	-	-
Suburban	1.38* (0.99–1.92)	-	-
Town	0.69** (0.49–0.99)	-	-
Village	0.30*** (0.20–0.47)	-	-
Region of residence			
North	1 [Reference]	1 [Reference]	1 [Reference]
South	1.22 (0.94–1.58)	1.29* (0.97–1.70)	0.90 (0.43–1.88)
Socioeconomic status			
Employment			
Unemployed	1 [Reference]	1 [Reference]	1 [Reference]
Employed	0.89 (0.64–1.25)	0.93 (0.65–1.33)	0.83 (0.24–2.93)
Annual household income (Yuan)			
0–8000	1 [Reference]	1 [Reference]	1 [Reference]
8001–15,000	1.05 (0.67–1.65)	1.04 (0.62–1.74)	1.11 (0.42–2.94)
15,001–25,000	1.04 (0.67–1.63)	1.11 (0.67–1.84)	0.61 (0.20–1.91)
Over 25,000	1.40 (0.92–2.14)	1.49* (0.93–2.39)	1.24 (0.45–3.43)
Education			
Illiterate	1 [Reference]	1 [Reference]	1 [Reference]
Primary school	1.92** (1.14–3.25)	1.81** (1.02–3.22)	3.00 (0.72–12.52)
Middle school	2.53*** (1.51–4.23)	2.46*** (1.41–4.30)	2.38 (0.54–10.56)
High school or above	3.72*** (2.22–6.22)	3.25*** (1.86–5.68)	9.33*** (2.20–39.55)
Health behavior			
Smoking status			
Nonsmoker	1 [Reference]	1 [Reference]	1 [Reference]
Smoker	0.84 (0.60–1.19)	0.89 (0.62–1.29)	0.55 (0.20–1.48)
Alcohol consumption			
No drinking	1 [Reference]	1 [Reference]	1 [Reference]
Drinking	1.16 (0.86–1.58)	1.21 (0.87–1.67)	0.83 (0.29–2.38)
Health-related variables			
BMI categories			
Underweight	1 [Reference]	1 [Reference]	1 [Reference]
Normal weight	1.26 (0.57–2.78)	1.19 (0.50–2.82)	1.95 (0.20–19.11)
Overweight	1.41 (0.63–3.15)	1.41 (0.59–3.38)	1.20 (0.12–12.47)
Obese	1.31 (0.55–3.11)	1.29 (0.51–3.30)	0.85 (0.57–12.83)
Current health status (self-report)			
Very good	1 [Reference]	1 [Reference]	1 [Reference]
Good	1.48** (1.02–2.15)	1.49* (0.99–2.24)	1.28 (0.47–3.51)
Bad	1.10 (0.73–1.66)	1.01 (0.65–1.58)	1.63 (0.58–4.77)
Very bad	0.61 (0.29–1.30)	0.61 (0.27–1.37)	0.62 (0.06–5.97)
Health insurance coverage			
No	1 [Reference]	1 [Reference]	1 [Reference]
Yes	1.77*** (1.35–2.33)	1.53*** (1.14–2.06)	4.07*** (1.92–8.60)

*Abbreviations: CI* confidence interval, *BMI* Body Mass Index; —, not applicable

*p < .10

**p < .05

***p < .01

The odds ratios and 95% CI of physical activity with and without confounders have been compared. After removing BMI and current health status (self-reported) from multivariate models, odds ratios and 95% CI of physical activity among the whole sample, urban residents, and rural residents did not dramatically change. The result is available upon request.

## Discussion

This study examined the association between LTPA preference and LTPA behavior among a longitudinal sample of adults who participated in the China Health & Nutrition Survey in both 2004 and 2011. The longitudinal sample allowed us to examine the effects of participants’ LTPA preference in 2004 on their LTPA behavior in 2011 by setting the temporal order. Overall, LTPA was not commonly practiced in our sample, which is consistent with previous studies [7, 31]. In addition, the results reveal that LTPA preference was a significant predictor of LTPA behavior, which supports our original hypothesis, but only among the whole sample and urban residents, and not among rural participants.

Preferring LTPA was strongly and positively correlated with performing LTPA. This is not surprising in light of existing research on the causal relationship between food preference and food intake, and between sedentary activity preference and sedentary activity behavior [1–6]. Those studies specifically show the predicting value of food preference on actual food intake, and causal relationships between sedentary activity preference and lower physical activity level. This study confirms that measuring participants’ preference on LTPA provides evidence of their actual activity level. The results are consistent with the original hypothesis, although future multiple genetic analyses and longitudinal analyses might provide more information on the pathway from preference to behavior.

The urban-rural difference was observed in our study. LTPA was substantially higher among urban residents than their rural counterparts, and urban participants were more likely to prefer LTPA. This could be explained by previous research [11–13], which shows that urban adults were more physically active during leisure time than their rural counterparts, while rural residents were more involved in TV watching. In addition, the significant association between LTPA preference and activity was found in urban residents, but not in rural participants. A possible explanation could be that the urban residents had more access to facilities and more spaces for physical activity during leisure time than those in the rural areas [32, 33]. Therefore, it would not be surprising to see that rural residents who prefer LTPA might find it impractical to do exercise, thus leading to lower activity level. Alternatively, the unadjusted confounding effects (such as occupational physical activity) might contribute to the association difference. For instance, rural residents may be busy with physical farm-work, and this made leave them with little leisure time and less perceived need for physical activities. Both of these factors could result in an observation of diminished association in rural residents.

The confounding effects of BMI and current health status (self-report) had been assessed by sensitivity analysis, given that a number of existing studies suggest the two variables have a significant impact on physical activity [34–37]. The difference between before and after removing them was however not substantial, suggesting that the causal relationship between LTPA preference and behavior observed in the study was not confounded by the two variables.

This study has several limitations. First, there was no qualitative data on physical activity related to beliefs about the purpose of performing physical activity, the perceived usefulness of physical activity, the impact physical activity has on other health behaviors, and so forth. Thus, though physical activity preference predicts physical activity behavior, one cannot fully disentangle why and how this is so. Furthermore, the use of self-reported data can potentially introduce recall bias, especially for variables based on participants’ long-term memory; for example, duration of physical activity [38]. In addition, the questions on LTPA preference and behavior were based on different types of activity, which may further obscure their association, especially among rural residents for whom a significant correlation was not found. For example, individual sports activities such as soccer and tennis were surveyed in the behavior section, but sport in general was queried in the preference section. Furthermore, regardless of the substantial statistical significance in the Spearman correlation coefficients, the associations in the analyses were not very strong. This may also be due to the discrepancy of types of activity surveyed regarding LTPA preference and actual behavior. Finally, this study was based on a sample in China, where cultural norms and patterns of LTPA may be unique to the Chinese context, and therefore may limit the ability to generalize our findings to other countries.

Despite these limitations, this population-based study is unique in establishing the causal relationship between LTPA preference and behavior. To the best of our knowledge, this is the first scientific attempt to examine the predictive value of preference for LTPA. In addition, our study used longitudinal data, which allows for inferring causality.

### Applications

The major finding of this study underscoring that LTPA preference among adults in China is a significant predictor of reported physical activity behavior, has some implications for health promotion researches and interventions. Measuring LTPA preferences may provide an alternative to the actual physical activity assessment, considering the possible recall bias incurred by traditional surveys. Specializing in changing preference for LTPA through comprehensive health promotion interventions such as community involvement in modeling healthful physical activity is also more likely to have a positive impact than direct and simple education on knowledge. Our findings regarding urban and rural differences may also suggest future interventions be tailored according to intervention settings. Health interventions in rural areas especially may also focus more on increasing residents’ access to physical activity facilities and on investing more in infrastructure development, beyond preference education.

## Conclusions

The study has proved the predictive value of LTPA preference on actual LTPA behavior. This may provide researchers an alternative to the traditional physical activity assessment. Changing people’s LTPA preference to increase LTPA may be helpful in chronic disease prevention and control in China, and may be able to be applied to other countries and contexts. In addition, health interventions in rural areas may increase residents’ access to LTPA facilities.

## Appendix

**Table 3:** Comparison of samples with and without those did not express physical activity preference

	Sample without those did not express activity preference		Sample with those did not express activity preference	
Variables	Number	Mean/Percentage (SD)	Number	Mean/Percentage (SD)
Demographics				
Age	2645	40.49 (12.84)	5685	39.06 (12.25)
Sex				
Male	1253	47.4	2551	44.9
Female	1392	52.6	3134	55.1
Ethnicity				
Han	2391	90.4	4983	87.7
Others	254	9.6	702	12.3
Marital status				
Never married	108	4.1	265	4.7
Married	2448	93.0	5237	92.5
Divorced	16	0.6	30	0.5
Widowed	60	2.3	130	2.3
Community types				
Urban	482	18.2	608	10.7
Suburban	640	24.2	1099	19.3
Town	526	19.9	843	14.8
Village	997	37.7	3135	55.1
Region of residence				
North	1268	47.9	2489	43.8
South	1377	52.1	3196	56.2
Socioeconomic status				
Employment				
Unemployed	570	21.6	872	15.4
Employed	2072	78.4	4807	84.6
Annual household income (Yuan)				
0–8000	520	19.9	1371	24.1
8001–15,000	643	24.6	1517	26.9
15,001–25,000	601	23.0	1294	23.0
Over 25,000	850	32.5	1449	25.7
Education				
Illiterate	464	17.6	1298	22.9
Primary school	593	22.5	1511	26.6
Middle school	781	29.6	1709	30.1
High school or above	799	30.3	1154	20.3
Health behavior				
Smoking status				
Nonsmoker	1781	67.4	3850	67.8
Smoker	861	32.6	1827	32.2
Alcohol consumption				
No drinking	1726	65.3	3850	67.7
Drinking	919	34.7	1835	32.3
Health-related variables				
BMI categories				
Underweight	96	3.8	289	5.4
Normal weight	1313	52.4	3044	56.8
Overweight	855	34.1	1571	29.3
Obese	241	9.6	458	8.5
Current health status (self-report)				
Very good	433	16.4	728	12.8
Good	1185	44.9	2581	45.5
Bad	872	33.0	1981	35.0
Very bad	151	5.7	378	6.7
Health insurance coverage				
No	1699	64.5	4233	74.9
Yes	936	35.5	1420	25.1

*Abbreviation: SD* standard deviation; —, not applicable

## Abbreviations

BMI: Body mass index
CHNS: China Health and Nutrition Survey
CI: Confidence interval
LTPA: Leisure time physical activity
MET-h: Metabolic equivalent task-hours
OA-ESI: Older Adult Exercise Status Inventory study
OR: Odds ratio
SD: Standard deviation
SES: Socio-economic status
SPSS: Statistical package for the social sciences
WHO: World Health Organization

## References

[1] Drewnowski A, Hann C (1999). Food preferences and reported frequencies of food consumption as predictors of current diet in young women. Am J Clin Nutr.

[2] Duffy VB, Lanier SA, Hutchins HL, Pescatello LS, Johnson MK, Bartoshuk LM (2007). Food preference questionnaire as a screening tool for assessing dietary risk of cardiovascular disease within health risk appraisals. J Am Diet Assoc.

[3] Harvey-Berino J, Hood V, Rourke J, Terrance T, Dorwaldt A, Secker-Walker R (1997). Food preferences predict eating behavior of very young Mohawk children. J Am Diet Assoc.

[4] Ricketts C (1997). Fat preferences, dietary fat intake and body composition in children. Eur J Clin Nutr.

[5] Salmon J, Owen N, Crawford D, Bauman A, Sallis JF (2003). Physical activity and sedentary behavior: a population-based study of barriers, enjoyment, and preference. Health Psychol.

[6] Temple VA (2007). Barriers, enjoyment, and preference for physical activity among adults with intellectual disability. Int J Rehabil Res.

[7] Sallis JF, Alcaraz JE, McKenzie TL, Hovell MF (1999). Predictors of change in children’s physical activity over 20 months: variations by gender and level of adiposity. Am J Prev Med.

[8] Bauman A, Phongsavan P, Schoeppe S, Owen N (2006). Physical activity measurement-a primer for health promotion. Promot Educ.

[9] Prince SA, Adamo KB, Hamel ME, Hardt J, Gorber SC, Tremblay M (2008). A comparison of direct versus self-report measures for assessing physical activity in adults: a systematic review. Int J Behav Nutr Phys Act.

[10] Zhu W, Chi A, Sun Y (2016). Physical activity among older Chinese adults living in urban and rural areas: a review. J Sport Health Sci.

[11] Bauman A, Ma G, Cuevas F (2011). Cross-national comparisons of socioeconomic differences in the prevalence of leisure-time and occupational physical activity, and active commuting in six Asia-Pacific countries. J Epidemiol Community Health.

[12] Du S, Lu B, Zhai F, Popkin BM (2002). A new stage of the nutrition transition in China. Public Health Nutr.

[13] Yang G, Kong L, Zhao W (2008). Emergence of chronic non-communicable diseases in China. Lancet.

[14] Zhang J (2012). The impact of water quality on health: evidence from the drinking water infrastructure program in rural China. J Health Econ.

[15] Thomas D, Frankenberg E, Smith JP (2001). Lost but not forgotten: attrition and follow-up in the Indonesia family life Survey. J Hum Resour.

[16] Zhao Z. Earnings instability and earnings inequality in urban China: 1989–2006. *Available at SSRN 1136432.* 2007.

[17] Chen H (2013). Parent-child fat intake correlation in China, explannation from social cognitive theory.

[18] Wilson KS, Spink KS (2009). Social influence and physical activity in older females: does activity preference matter?. Psychol Sport Exerc.

[19] Ainsworth BE, Haskell WL, Whitt MC (2000). Compendium of physical activities: an update of activity codes and MET intensities. Med Sci Sports Exerc.

[20] Brien-Cousins SO (1996). An older adult exercise status inventory: reliability and validity. J Sport Behav.

[21] Chogahara M (1999). A multidimensional scale for assessing positive and negative social influences on physical activity in older adults. J Gerontol Ser B Psychol Sci Soc Sci.

[22] Chen Y, Ebenstein A, Greenstone M, Li H (2013). Evidence on the impact of sustained exposure to air pollution on life expectancy from China’s Huai River policy. Proc Natl Acad Sci.

[23] Sharadha SO, Punithavathi N, Renuka Devi TK (2016). Better predictor of adverse pregnancy outcome: Asian or WHO international Cutoff? A single-Centre prospective study. J Obstet Gynaecol India.

[24] Sai-Chuen Hui S (2016). Sensitivity and specificity of WHO-BMI Criteria for obesity of Asian youth: the Asia-fit study: 615 board# 5 June 1, 1: 00 PM-3: 00 PM. Med Sci Sports Exerc.

[25] Park SK (2016). Effect of overweight and obesity (defined by Asian-specific Cutoff Criteria) on left ventricular diastolic function and structure in a general Korean population. Circ J.

[26] Mukhopadhyay S, Dutta D (2017). Is it justified to have a lower BMI Cutoff for metabolic surgery for Asians with type 2 diabetes?. Obes Surg.

[27] Nishida C (2004). Appropriate body-mass index for Asian populations and its implications for policy and intervention strategies. Lancet.

[28] Zhou J, Kessler AS, Su D (2016). Association between daytime napping and chronic diseases in China. Am J Health Behav.

[29] Chen CM, et al. Criteria of weight for adults. In: Health industrial standard of the People's Republic of China. China's State Family Planning Commission; 2013. http://www.moh.gov.cn/ewebeditor/uploadfile/2013/08/20130808135715967.pdf. Accessed 10 May 2017.

[30] SPSS (2012). [computer program]. Version 21.0.

[31] Ng SW, Norton EC, Popkin BM (2009). Why have physical activity levels declined among Chinese adults? Findings from the 1991-2006 China health and nutrition surveys. Soc Sci Med.

[32] Loucaides CA, Chedzoy SM, Bennett N (2004). Differences in physical activity levels between urban and rural school children in Cyprus. Health Educ Res.

[33] Sheu-jen H, Wen-chi H, Patricia AS, Jackson PW (2010). Neighborhood environment and physical activity among urban and rural schoolchildren in Taiwan. Health Place.

[34] Tian Y (2016). BMI, leisure-time physical activity, and physical fitness in adults in China: results from a series of national surveys, 2000–14. Lancet Diabetes Endocrinol.

[35] Kirwan LB, Johnstone AM, Fyfe CL. An evaluation of BMI and the Healthy Working Lives Initiative on workplace physical activity levels (the NeuroFAST study). Proc Nutr Soc. 2016;75(OCE3):150.

[36] Meshe OF (2016). The relationship between physical activity and health status in patients with chronic obstructive pulmonary disease following pulmonary rehabilitation. Disabil Rehabil.

[37] Mosallanezhad, Z, et al. A structural equation model of the relation between socioeconomic status, physical activity level, independence and health status in older Iranian people. Arch Gerontol Geriatr*.* 2017. 10.1016/j.archger.2017.01.004 28131051

[38] Hassan E (2006). Recall bias can be a threat to retrospective and prospective research designs. Int J Epidemiol.

